# Alexithymia and eating disorders: a critical review of the literature

**DOI:** 10.1186/2050-2974-1-21

**Published:** 2013-06-18

**Authors:** Matilda E Nowakowski, Traci McFarlane, Stephanie Cassin

**Affiliations:** 1 Department of Psychology, Ryerson University, 350 Victoria Street, Toronto, Ontario M5B 2K3, Canada; 2 Eating Disorder Program, The Toronto General Hospital, 200 Elizabeth Street, Toronto, Ontario M5G 2C4, Canada

**Keywords:** Alexithymia, Eating disorders, Emotion regulation

## Abstract

Alexithymia is characterized by difficulties identifying feelings and differentiating between feelings and bodily sensations, difficulties communicating feelings, and a concrete cognitive style focused on the external environment. Individuals with eating disorders have elevated levels of alexithymia, particularly difficulties identifying and describing their feelings. A number of theoretical models have suggested that individuals with eating disorders may find emotions unacceptable and/or frightening and may use their eating disorder symptoms (i.e., restricting food intake, bingeing, and/or purging) as a way to avoid or cope with their feelings. The current critical review synthesizes the literature on alexithymia and eating disorders and examines alexithymia levels across eating disorders (i.e., anorexia nervosa, bulimia nervosa, and eating disorder not otherwise specified), the role of alexithymia in binge eating disorder, and the influence of alexithymia on the development of eating disorders as well as treatment outcome. The clinical implications of the research conducted to date and directions for future research are discussed.

## Alexithymia and eating disorders: a critical review

The concept of alexithymia was first identified by Sifneos [1], who described a set of characteristics observed in psychosomatic patients that included difficulties identifying feelings and differentiating between feelings and bodily sensations, difficulties communicating feelings, lack of fantasy, and a concrete cognitive style focused on the external environment.

The majority of studies focusing on eating disorders have found higher levels of alexithymia in individuals with eating disorders and disturbed eating compared to healthy controls [2-8]. When the individual characteristics of alexithymia are examined, individuals with eating disorders have specific deficits in identifying and communicating their feelings.

A number of theories have been proposed regarding the underlying role of emotions in eating disorders. It has been suggested that patients use maladaptive eating behaviors (e.g., bingeing, purging, dietary restriction) and excessive exercise as a way to avoid or cope with their emotions [9-11]. Specifically, an early childhood environment in which emotions are viewed as unacceptable or frightening leads to the development of the belief that emotions are bad and should not be experienced or expressed. These beliefs become activated each time that an emotion is experienced, which then leads to a secondary emotion (i.e., an emotion in response to another emotion) such as shame, guilt, or disgust about experiencing an emotion. These secondary emotions increase the patient’s distress and decrease his/her coping abilities, thus leading to engagement in eating disorder behaviors in an attempt to avoid or cope with the emotion [12].

Although a robust body of literature illustrates that alexithymia levels, especially difficulties identifying and communicating feelings, are elevated in individuals with eating disorders, there remains much debate surrounding a number of issues, including: 1) whether alexithymia levels vary across eating disorder diagnoses; 2) whether alexithymia is a trait or state variable; and 3) whether current treatments for eating disorders effectively target alexithymia. To date, no review paper has synthesized the research on alexithymia in eating disorders. The purpose of the current paper is to synthesize the research addressing the aforementioned questions in order to increase theoretical knowledge of alexithymia and eating disorders and enhance clinical interventions.

## Methods

**Figure 1:**
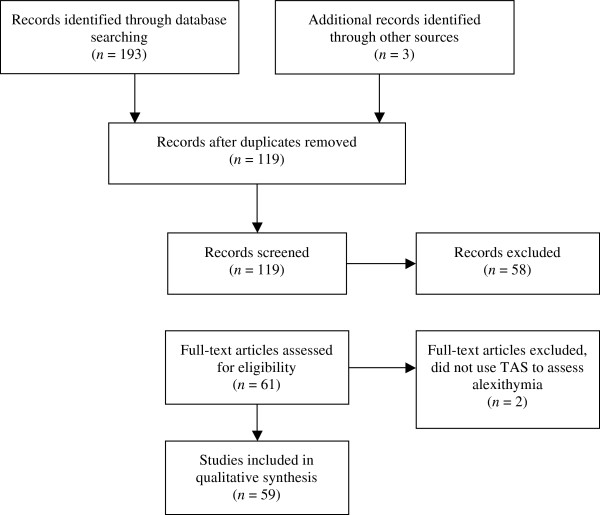
Diagram illustrating the process of selecting articles for the critical review.

A systematic review of the literature on alexithymia and eating disorders was conducted in September 2012 using PsycInfo. The following search terms were used in four literature searches: “alexithymia” and “anorexia nervosa”, “bulimia nervosa”, “eating disorders”, or “binge eating disorder”. The literature searches were limited to articles focusing on adolescents and adults that were published in peer-reviewed journals after January 1, 1988, and were written in English. We limited our search to articles published in 1988 or later as 1988 is the publication year of the first version of the Toronto Alexithymia Scale [13]. Our systematic review was limited to studies using the Toronto Alexithymia Scale, the most widely used and well-established measure of alexithymia, in order to facilitate comparisons across studies. This initial search identified 198 articles. The reference list of each article was subsequently examined for relevant articles that may have been omitted by PsycInfo, which resulted in 3 more articles being identified. After eliminating duplicates, 119 articles were identified. The article abstracts were then reviewed and coded for the following criteria: 1) one of the three versions of the Toronto Alexithymia Scale was used to assess alexithymia; 2) the study included clinical or non-clinical adolescent and/or adult participants with disturbed eating; 3) the study focused on identifying and/or explaining group differences in alexithymia levels; and 4) the study had a quantitative experimental design. Consistent with the continuum perspective of eating behaviors [14], studies including both clinical and non-clinical samples were included in the current review to provide a more complete picture of the research on alexithymia and disordered eating. This initial review of the abstracts resulted in 58 articles being excluded (5 did not use one of the versions of the TAS, 23 did not focus on eating disorders, 5 did not have a quantitative experimental design, and 27 did not address group differences or the role of alexithymia in eating disorders). After reviewing the full articles, two more articles were excluded as they did not use one of the three versions of the TAS to assess alexithymia levels. This resulted in 59 articles being included in the qualitative synthesis of the current review paper. The process of article selection is outlined in Figure 1.

### Measurement of alexithymia

The Toronto Alexithymia Scale (TAS) is the most widely used self-report measure of alexithymia. It was originally developed by Bagby and colleagues in 1988 and has since undergone two revisions (TAS-R and TAS-20). The original version of the TAS consisted of 26 items that were reduced to 4 factors: 1) difficulties identifying feelings and differentiating between feelings and bodily sensations (e.g., “I have feelings that I can’t quite identify”); 2) difficulties describing feelings (e.g., “People tell me to describe my feelings more”); 3) lack of fantasy (e.g., “I daydream rarely”); and 4) externally-oriented thinking (e.g., “Knowing the answers to problems is more important than knowing the reasons for the answers”) [13]. The psychometric properties of the original TAS had a number of limitations, including susceptibility to social desirability response biases and low item-total correlations for the lack of fantasy factor, which lead to the creation of a 23-item revised version (TAS-R) [15]. However, the TAS-R [15] had an unstable factor structure such that the difficulties identifying and describing feelings factors collapsed into one single factor. As well, the majority of the lack of fantasy items still showed poor item-total correlations. As a result, in the second revision of the TAS (TAS-20) [16], the items for the lack of fantasy factor were eliminated and the items for the identifying and describing feelings factors were modified. The final scale consisted of 20 items and 3 factors: 1) difficulties identifying feelings and differentiating between feelings and bodily sensations (e.g., “I am often confused about what emotion I am feeling”); 2) difficulties describing feelings (e.g., “It is difficult for me to find the right words for my feelings”); and 3) externally-oriented thinking (e.g., “I prefer to just let things happen rather than to understand why they turned out that way”). Based on empirically determined cut-off scores, individuals who score 61 or higher on the TAS-20 are identified as alexithymic [16]. Therefore, alexithymia can be assessed on both a continuous and a categorical basis.

The majority of studies using patient and non-patient samples have replicated the 3-factor structure of the TAS-20 [16-20], although some studies using patient samples have found a 2-factor structure, with the difficulties identifying and difficulties describing feelings factors comprising a single factor [21,22]. The internal consistencies for the difficulties identifying feelings and difficulties describing feelings factors have been in the acceptable range across numerous studies [17-20]. In contrast, the internal consistency for the externally oriented thinking factor has been low [17-20]. Studies using non-clinical samples have demonstrated good test-retest reliability for all factors, although no studies have examined a time-span longer than 3 months [16,17,19]. Thus, although the TAS-20 appears to be a psychometrically sound measure, it is important to note its psychometric limitations, notably the low internal consistency for the externally oriented thinking factor and the absence of studies investigating its test-retest reliability in clinical samples.

### Is alexithymia elevated in individuals with eating disorders compared to controls?

To date, 24 studies have investigated whether alexithymia is elevated in individuals with eating disorders using one of the three versions of the Toronto Alexithymia Scale. See Table 1 for a summary of the studies. Across studies, significantly more eating disorder patients are categorized as alexithymic compared to healthy community controls [4,5,8,23-34]. When examining alexithymia as a continuous measure, studies have shown that individuals with eating disorders have higher total alexithymia scores as well as higher scores on two out of three factors: 1) Difficulties identifying feelings and differentiating between feelings and bodily sensations, and 2) Difficulties describing feelings [2,4,5,24-28,30-43]. Studies have generally not reported differences between individuals with eating disorders and healthy community controls on externally oriented thinking (but see [30,42]), although this may be a reflection of the poor internal consistency for the externally oriented thinking factor. In sum, a robust body of research illustrates that individuals with eating disorders show specific deficits in identifying and describing affective states compared to healthy community controls.

**Table 1:** Studies investigating alexithymia in clinical samples of patients with anorexia nervosa, bulimia nervosa, and binge eating disorder

**Authors**	**Year**	**Diagnostic Tool**	**Sample Characteristics**	**Alexithymia Measure**	**Results**
Beales & Dolton	2000	EDI	Females, outpatient, 17–46 years old, AN-R (26), BN (29), recovered (24)	TAS-20	Alexithymic: BN (83%), AN-R (65%), recovered (33%)
Berthoz et al.	2007	MINI	Females, AN-R (50), AN-B/P (5), BN (20)	TAS-20	No group differences on TAS-20 scores
Bourke et al.	1992	DSM-III-R	Females, outpatient, AN-R (30), AN-B/P (18), HC (30)	TAS	Alexithymic: AN (77.1%), HC (6.7%); TAS Total: AN > HC
Bydlowski et al.	2005	DSM-IV	Females, outpatient, AN-R (18), AN-B/P (15), BN (37), HC (70)	TAS-20	Alexithymic: EDs (60%), HC (30%); TAS-20 Total: EDs > HC, no differences between EDs, group differences disappeared when controlling for depression but not when controlling for anxiety
Carano et al.	2006	DSM-IV	Males and females, outpatient, obese + BED (101)	TAS-20	Alexithymic: 39.6%
Corcos et al.	2000	DSM-IV	Males and females, outpatient, AN-R (21), AN-B/P (11), BN (32), HC (74)	TAS-20	TAS Total: EDs > HC, AN > BN, group differences between AN and BN disappeared when controlling for depression
Cochrane et al.	1993	DSM-III-R	Females, outpatient, AN (19), BN (52), AN + BN (18), EDNOS (25), HC (370)	TAS	TAS Total Score: EDs > HC, no differences between EDs
de Groot	1995	DSM-III-R	Females, day hospital program, BN (31), HC (female nurses; 20)	TAS	Alexithymic: BN (61.3%), HC (5%), TAS Total, DIF, DDF: BN > HC, group differences for TAS Total and DIF remained when controlling for depression
De Panfilis et al.	2003	DSM-IV	Females, outpatient, AN-R (4), AN-B/P (12), BN (20), BED (28), HC (68)	TAS-20	Alexithymic: EDs (14.4%), HC (1.6%); TAS-20 Total, DIF: EDs > HC
de Zwaan et al.	1995	DSM-IV	Males and females, outpatient, obese + BED (83), obese + no BED (99)	TAS	TAS Total: no group differences
Deborde et al.	2008	MINI	Females, AN-R (10), AN-B/P (7), BN (30), HC (253)	TAS-20	Alexithymic: EDs (53.2%), HC (13.83%)
Eizaguirre et al.	2004	DSM-IV	Females, AN-R (25), AN-BP (44), BN (82), HC (43)	TAS-20	Alexithymic: AN-R (56%), AN-B/P (68.2%), BN (47%), HC (2.3%), TAS-20 Total, DIF, DDF, EOT: EDs > HC, DIF: AN-B/P > BN, AN-R, EOT: BN > AN-B/P, group differences disappeared when controlling for depression and anxiety
Gilboa-Schechtman et al.	2006	DSM-IV	Females, outpatient, AN (20), BN (20), HC (20)	TAS-20	TAS-20 DIF, DDF: EDs > HC, DDF scores: AN > BN, group differences disappeared when controlling for distress
Guttman et al.	2002	DSM-III-R	Females, outpatient, AN-R (34), HC (33)	TAS-20	TAS-20 Total, DIF, DDF: AN-R > HC
Jimerson et al.	1994	DSM-III-R	Females, outpatient, BN (20), HC (20)	TAS	Alexithymic: BN (40%), HC (0%); TAS Total, DIF, DDF: BN > HC, group differences disappeared for DDF when controlling for anxiety and depression
Lawson et al.	2008	DSM-IV	Females, outpatient, AN (11), BN (21), EDNOS (38)	TAS-20	TAS-20 Total, DIF, DDF: no group differences
Montebarocci et al.	2006	DSM-IV	Females, outpatient, AN-R (18), BN (16), HC (18)	TAS-20	Alexithymic: AN-R (50%), BN (56%), HC (22%); TAS-20 Total: AN-R, BN > HC, DIF: BN, AN-R > HC, EOT: AN-R > BN, HC, group differences disappeared when controlling for anxiety and depression
Nandrino et al.	2006	DSM-IV	Females, inpatient, AN-R (25), HC (25)	TAS-20	TAS-20 Total: AN-R > HC
Parling et al.	2010	DSM-IV	Females, outpatient, AN (35), HC (34)	TAS-20	TAS-20 DIF, DDF: AN > HC, group differences disappeared when controlling for anxiety and depression
Pascual et al.	2011	Self-report questionnaires	Females, BN-P (17), BN-NP (17), nonspecific AN (22), nonspecific BN (42)	TAS-20	DDF: nonspecific AN > nonspecific BN, BN-NP
Pinaquy et al.	2003	DSM-IV	Females, obese + BED (40), obese + no BED (129)	TAS-20	TAS-20 Total, DIF, DDF: obese + BED > obese + no BED
Rastam et al.	1997	DSM-III-R	Males and females, AN-R (48), HC (51)	TAS-20	Alexithymic: AN-R (23%), HC (6%)
Schmidt et al.	1993	DSM-III-R	Females, outpatient, BN (93), AN-R (55), AN-B/P (25), HC (48 females and 47 males)	TAS	Alexithymic: AN-R (56%), AN-B/P (48%), BN (50%), HC (17.8%); TAS Total: All EDs > HC, AN-R > BN
Sexton et al.	1998	DSM-IV	Females, inpatients, AN-R (15), AN-B/P (16), BN (22), HC (14)	TAS	TAS Total: AN-R, AN-B/P > HC; DIF, DDF: AN-B/P, BN > HC; DDF: AN-R > BN; after controlling for depression: TAS Total: AN-R > HC; DDF: AN-R > BN, HC
Sureda et al.	1999	DSM-III-R	Females, outpatient, BN (35), HC hospital staff (35)	TAS-20	Alexithymic: BN (51.4%), HC (6.3%); TAS-20 Total, DIF, DDF: BN > HC
Speranza et al.	2005	DSM-IV	Females, outpatient, AN-R (105), AN-B/P (49), BN (89), HC (279)	TAS-20	TAS-20 DIF, DDF: all EDs > HC, when controlling for depression; DIF: BN > HC; DDF: AN-R > HC
Taylor et al.	1996	DSM-III-R	Females, outpatient, AN (48), HC hospital staff (30), HC undergraduates (234)	TAS-20	Alexithymic: AN (68.8%), HC hospital staff (3.3%), HC undergraduates (13.7%); TAS-20 Total, DIF, DDF: AN > all HC groups
Torres et al.	2011		Females, outpatient, AN-R (52), AN-B/P (28), HC (80)	TAS-20	TAS-20 DIF, DDF, EOT: AN > HC
Troop et al.	1995	DSM-III-R	Females, outpatient, AN-R (29), AN-B/P (15), BN (83), HC (79)	TAS	TAS Total, DIF: EDs > HC; lack of fantasy: AN-R > BN, HC
Zeeck et al.	2011	DSM-IV	Females, outpatient, BED (20), obese (23), HC (20)	TAS-20	TAS-20 Total: BED > obese, HC
Zonnevijlle-Bender et al.	2002	DSM-IV	Females, outpatient, AN (16), BN (8), EDNOS (6), HC (33)	TAS-20	Alexithymic: AN (60%), BN (50%), EDNOS (14%), HC (10%), TAS-20 Total: EDs > HC
Zonnevijlle-Bender et al.	2004	DSM-IV	Females, AN-R (48), psychiatric control (anxiety and depression; 21), HC (48)	TAS-20	Alexithymic: AN (60%), psychiatric control (52%), HC (27%); TAS-20 Total, DIF, DDF: AN > HC

EDI = Eating Disorder Inventory [44]; MINI = The Mini-International Neuropsychiatric Interview [45]; DSM-III-R = Diagnostic Statistical Manual of Mental Disorders 3^rd^ Edition Revised [46]; DSM-IV = Diagnostic Statistical Manual of Mental Disorders 4^th^ Edition [47]; AN-R = Anorexia Nervosa – Restricting Type; AN-B/P = Anorexia Nervosa – Bingeing/Purging Type; BN = Bulimia Nervosa; BED = Binge Eating Disorder; HC = healthy control; TAS = Toronto Alexithyma Scale 26-item Version [13]; TAS-20 = Toronto Alexithymia Scale 20-item Version [16]; TAS Total = Toronto Alexithymia Scale Total Score; DIF = Toronto Alexithymia Scale Difficulties Identifying Feelings factor; DDF = Toronto Alexithymia Scale Difficulties Describing Feelings factor, EOT = Toronto Alexithymia Scale Externally Orienting Thinking factor.

### Is alexithymia elevated in nonclinical samples with disturbed eating?

Studies conducted with nonclinical undergraduate samples have found that higher levels of eating disorder behaviors (e.g., bingeing, purging, dietary restriction, excessive exercise) are associated with higher scores on the difficulties identifying feelings and difficulties describing feelings factors of the TAS/TAS-R/TAS-20 [3,6,7,48-55]. In general, no relations have been found between eating disorder behaviors and the externally oriented thinking factor on the TAS/TAS-R/TAS-20. This pattern of results is consistent with the pattern found for clinical samples, providing further support that individuals with eating disturbances experience difficulties with identifying and communicating emotions.

### Is the relation between alexithymia and eating disorders accounted for by general distress?

Clinical studies investigating the relation between eating disorder behaviors and alexithymia when controlling for general distress (i.e., anxiety and depression) have generated mixed results. Some studies have found that group differences on difficulties identifying and describing feelings disappear when general distress is controlled for [4,25,30,36,39]. For instance, using a female sample of 40 outpatients with DSM-IV eating disorders (i.e., AN-R, AN-B/P, and BN), 19 of who also had comorbid major depressive disorder, and a sample of 20 healthy control females, Gilboa-Schechtman and colleagues [36] found that patients with eating disorders reported significantly higher scores on the TAS-20 difficulties identifying and difficulties describing feelings factors compared to the healthy controls. However, group differences disappeared when analyses controlled for both depression and anxiety.

In contrast, other studies have found that individuals with eating disorders continue to exhibit elevated alexithymia levels even when controlling for general distress [5,24,27,40,56]. For instance, controlling for depression levels, Bourke and colleagues [24] found significantly higher total alexithymia scores, as measured by the TAS, in a sample of 48 female outpatients who met DSM-III-R diagnostic criteria for AN compared to 30 female controls. Similarly, in a sample of 20 female outpatients who met DSM-III-R criteria for BN and 20 matched female controls, patients with BN had significantly higher scores on the TAS difficulties identifying and describing feelings factors as well as the TAS total score compared to matched female controls. When anxiety and depression were controlled, group differences remained for the total alexithymia score and the difficulties identifying feelings factor [5]. A recent study by Rozenstein and colleagues [56] found that depression explained group differences for the TAS-20 total score and the difficulties identifying feelings and difficulties describing feelings factor scores for patients with AN-R but not for patients with BN and AN-B/P. Rozenstein and colleagues [56] suggested that different mechanisms might underlie difficulties with identifying and communicating emotions for patients who restrict versus patients who engage in bingeing and purging behaviors. Specifically, while depression may directly contribute to difficulties with identifying and expressing emotions in patients who restrict, it may be the increased impulsivity and affective instability that underlies the affective difficulties of patients with bingeing and purging behaviors.

Our review of the literature on the role of depression in explaining group differences on alexithymia levels partially supports Rozenstein and colleagues’ [56] proposal. Two of the studies that found that depression did not explain group differences in alexithymia levels focused solely on patients with BN [5,27]. Furthermore, although Eizaquirre and colleagues [4] found that group differences on alexithymia levels disappeared when controlling for both depression *and* anxiety, when only depression was controlled for, group differences between the control group and the AN-B/P and BN groups for the difficulties identifying feelings factor remained. It should be noted, however, that both Bydlowski and colleagues [25] and Gilboa-Schechtman and colleagues [36] failed to find any differences between patients who restricted and those who engaged in bingeing and purging behaviors after controlling for anxiety and depression levels. Further, using a sample of 53 female inpatients, Sexton and colleagues [40] found that after controlling for depression levels, only the AN-R group had significantly higher scores on the TAS total score and the difficulties communicating feelings factor compared to the control group.

As illustrated above, the findings for the role of anxiety and depression in accounting for group differences in alexithymia are mixed. There is a need for further research to clarify this pattern of findings. One of the limitations to date is that many of the studies in this area fail to distinguish between patients with AN-R and patients with AN-B/P. This differentiation is important in order to further test Rozenstein and colleagues’ [56] suggestions regarding the differential influence of depression on alexithymia in patients who restrict versus patients who engage in bingeing and purging behaviors. Furthermore, with the exception of a few studies [5,32,36,41], most studies do not report comorbid diagnoses for participants, thus failing to differentiate between subclinical depressive and anxiety symptoms and comorbid major depressive disorder and anxiety disorders. Given the potential identified role of depression and anxiety in alexithymia symptoms, this important information should be included in future studies in order to better understand the role of anxiety and depression in alexithymia.

### Do levels of alexithymia differ amongst the eating disorder diagnoses?

While some studies have not reported significant differences in alexithymia levels across the eating disorders [2,25,43,57,58], others have suggested that individuals with a diagnosis of AN experience higher levels of alexithymia [4,26,31,36,59]. In one of the first studies to investigate differences between eating disorders, Schmidt and colleagues [31] found that patients in the AN-R group reported significantly higher total alexithymia scores compared to patients in the AN-B/P and BN groups. Following these findings, a number of studies have examined group differences among eating disorders, further delineating alexithymia into its separate factors.

Three studies have found that individuals with AN report higher scores on the difficulties describing feelings factor compared to individuals with BN [36,40,59]. For instance, Pascual and colleagues [59] investigated group differences on the TAS-20 factors using a sample of 98 females divided into 4 groups: BN-purging type (*n* = 17), BN-non purging type (*n* = 17), nonspecific BN (met the majority of criteria for BN; *n* = 42), and nonspecific AN (met the majority of criteria for AN; *n* = 22). Participants in the nonspecific AN group reported significantly higher scores on the difficulties describing feelings factor compared to participants in the nonspecific BN group as well as the BN-non purging group. Participants in the BN-purging group had an intermediate profile such that their scores on difficulties describing feelings were lower than the nonspecific AN group but higher than the nonspecific BN group and the BN-non purging group. Similarly, Gilboa-Schechtman and colleagues [36] found that patients who met the DSM-IV criteria for AN reported significantly greater difficulties describing feelings compared to patients who met the criteria for BN. Based on the research to date, it appears that individuals with AN have elevated alexithymia levels, specific to difficulties expressing emotions, compared to individuals with BN.

### Is alexithymia elevated in binge eating disorder?

Studies focusing on BED samples have generally found an association between alexithymia and BED symptom severity [60-63], but see [64] for an exception. For instance, Carano and colleagues [60] found that difficulties identifying and describing feelings, as measured by the TAS-20, predicted the severity of BED in 101 patients who met DSM-IV diagnostic criteria for BED. Similarly, female obese patients who met DSM-IV criteria for BED had significantly higher total scores as well as higher scores on difficulties identifying and describing emotions compared to female obese patients who did not meet DSM-IV criteria for BED [61]. Consistent with the pattern of findings in AN and BN, individuals with BED have higher levels of alexithymia, specifically with regards to identifying and communicating emotions.

### What role does alexithymia play in the development of eating disorders?

To date, a paucity of research has specifically examined the potential etiological role of alexithymia in eating disorders, and the few studies that have been conducted have focused on childhood maltreatment and personality. Across studies using non-clinical undergraduate samples, childhood maltreatment has been indirectly associated with eating disorder symptoms through its relations with alexithymia and depression [65-69]. For instance, using a sample of 406 female undergraduates, Mazzeo and Espelage [67] found that high family conflict and low family cohesion were significantly associated with childhood physical and emotional abuse and neglect, and that the association between childhood maltreatment and disordered eating symptomatology was fully mediated by alexithymia and depression. This model was cross-validated and replicated using a second sample of 406 female undergraduate students [67]. Thus, in females, childhood physical and emotional abuse and neglect all increase the risk for depression and alexithymia, which in turn increase the risk for disordered eating symptoms. However, this pattern of results appears to vary with sex and ethnicity. For instance, in males, the relation between childhood maltreatment and bulimic symptoms appears to be mediated only by depression [69]. Furthermore, there appear to be differences between ethnic groups. Mazzeo and colleagues [68] found that the association of childhood maltreatment with eating disorder symptoms was mediated by self-reported anxiety, depression, and alexithymia in both African American and European American undergraduate female students. However, only the European American female sample had a positive relation between depression and alexithymia.

To date, only one study has investigated the relationship between personality factors and alexithymia in the development of eating disorders. Using a clinical sample of 49 patients with eating disorders, Ruggiero and colleagues [70] found that perfectionism mediated the relation between alexithymia and eating disorders, such that alexithymia increased the risk of perfectionism, which in turn increased the risk of developing an eating disorder.

In summary, the research to date suggests that low family cohesion and high levels of family conflict are associated with an increased risk for childhood physical and emotional abuse and neglect. In turn, childhood maltreatment increases the risk for depression and alexithymia, which both increase the risk for eating disorder behaviors. As noted above, sex and ethnic background might moderate these relationships, highlighting the importance of further examining sex and cultural differences in the eating disorder literature. In addition to familial factors, preliminary research also suggests a potential role of perfectionism in mediating the relation between alexithymia and eating disorders.

### Do alexithymia levels change following treatment?

To date, six studies, three of which have been uncontrolled, have examined the effects of treatment on alexithymia levels in patients with eating disorders. These studies have shown that alexithymia scores decrease significantly post-treatment in patients with AN, BN, and BED [27,71-75]. For instance, Becker-Stoll and Gerlinghoff [71] examined the effects of a 4-month day hospital treatment on alexithymia levels in a sample of 47 patients (AN = 18, BN = 25, and EDNOS = 4). The treatment program was multi-modal, including psychoeducation, cognitive behavior therapy, and interpersonal therapy. As well, during group therapy an emphasis was placed on encouraging patients to experience and communicate their emotions. Across diagnostic groups, patients who completed the treatment reported significant reductions in eating disorder symptomatology as well as significant reductions in TAS-20 total scores. When the TAS-20 subscales were examined, across diagnostic groups, only the difficulties identifying feelings factor showed a statistically significant reduction from pre- to post-treatment. Similarly, Storch and colleagues [75] found that following inpatient treatment for eating disorders (i.e., AN-R, AN-B/N, and BN) patients evidenced a significant increase in their ability to identify emotions.

Clyne and Blampied [73] found that an 11-week group treatment for BED with a strong focus on identifying, communicating and managing emotions lead to significant reductions in alexithymia and depression scores from pre-treatment to post-treatment, as well as from post-treatment to follow-up, in a sample of 11 females who met criteria for BED according to self-report questionnaires. Similarly, Ciano and colleagues [72] compared the effects of group-analytic therapy (*n* = 6) and group psychoeducation (*n* = 5) on alexithymia levels in patients with BED. The group-analytic therapy focused on identifying and accepting intrapsychic conflicts that underlie disordered eating behaviors while the psychoeducation focused on communication and assertiveness skills training, nutrition education, and problem solving. While patients in both treatments evidenced significant symptom improvement at post-treatment and at the 6- and 12-month follow-up assessments, only patients in the psychoeducation group also showed a significant improvement in their ability to describe their feelings.

In sum, evidence suggests that psychological treatment leads to a significant improvement in alexithymia scores from pre- to post-treatment. However, the question remains about the clinical significance of the decrease in alexithymia scores. That is, after patients receive treatment, are their alexithymia scores still elevated compared to controls? To date, only one controlled study has investigated this question. de Groot and colleagues [27] examined the effectiveness of a group day hospital treatment program in 31 female patients with a diagnosis of BN. Patients completed the TAS pre- and post-treatment, and a group of 20 nurses who served as the control group, completed the TAS at one time-point. de Groot and colleagues [27] found that, despite a statistically significant reduction in the alexithymia total score and the difficulties identifying and describing feelings factor scores, the patients still had significantly higher alexithymia scores at post-treatment compared to the controls. Furthermore, although there was a significant decrease in the percentage of patients who were categorized as alexithymic from pre- to post-treatment, the percentage of patients who were alexithymic at post-treatment was still significantly higher compared to the percentage of controls who were alexithymic. This is the only controlled treatment study to date that has investigated alexithymia levels pre and post-treatment, and unfortunately the implications of its results are limited by the biased control group (i.e., nurses working in the hospital). An examination of alexithymia scores across studies, however, shows that across non-treatment studies, the control group scores on the TAS and TAS-20 range from 53.0 [5] to 63.7 [43] and 43.10 [28] to 47.0 [41], respectively. An examination of the alexithymia scores in the treatment studies reviewed shows that, with the exception of the study by Becker-Stoll and Gerlinghoff [71], the post-treatment alexithymia scores are still higher compared to the control scores reported in the literature. Thus, the decrease in alexithymia scores from pre to post-treatment, although statistically significant, may not lower alexithymia levels to the values reported by controls.

Given the clear association between alexithymia and depression, a critical question is whether the changes in alexithymia scores from pre-treatment to post-treatment can be explained by changes in depression scores. Surprisingly, only two studies to date have examined this question. Sexton and colleagues [40] found that scores for difficulties identifying and difficulties describing feelings decreased following inpatient psychological treatment in a sample of 53 female inpatients with eating disorders (AN-R, AN-BP, and BN). However, this change was accounted for by a decrease in depressive symptoms. The authors did not describe the treatment protocol used, thus making it difficult to determine whether special emphasis was placed on helping patients to identify and communicate their feelings. In another study, de Groot and colleagues [27] found that in a sample of 31 females with a diagnosis of BN who completed a group day hospital treatment program, both alexithymia, as measured by the TAS, and depression decreased significantly from pre-treatment to post-treatment. Although there was a statistically significant relation between alexithymia and depression scores both at pre-treatment and at post-treatment, it was found that both patients who were depressed at pre-treatment but not at post-treatment, as well as patients who were not depressed at either time-point, had statistically significant decreases in alexithymia scores from pre-treatment to post-treatment. Thus, although alexithymia and depression may be significantly related, there is evidence that alexithymia is not simply a by-product of depression because there was improvement in alexithymia scores even when depression scores were stable.

Based on the research reviewed, it appears that psychological treatments for eating disorders lead to statistically significant decreases in alexithymia scores from pre-treatment to post-treatment, although in the majority of cases, the alexithymia scores are still elevated at post-treatment compared to controls. There is a need for more controlled treatment studies to better understand changes in alexithymia from pre- to post-treatment and the potential mediators of this change.

## Review

In summary, there is a robust body of literature illustrating that alexithymia levels, both from a continuous and a categorical perspective, are elevated in individuals with eating disorders compared to healthy controls. Furthermore, individuals with eating disorders have specific deficits in identifying and communicating emotions. One question that arises is whether alexithymia is a state or trait variable. There is strong evidence that alexithymia is not simply a by-product of eating disorder symptomatology. However, the evidence is mixed regarding whether alexithymia is independent of general distress. Although some studies have found that individuals with eating disorders no longer display elevated alexithymia scores once depression levels are controlled for [4,26,36,39], other studies have shown that individuals with eating disorders continue to have elevated alexithymia scores, even when depression levels are controlled for [5,24,27,40]. Furthermore, research evidence from treatment studies suggests that alexithymia levels decrease even when depression levels remain stable [27] and pharmacotherapy with antidepressants decreases depression levels but not alexithymia levels in individuals with high levels of both alexithymia and depression [31]. Lastly, genetic studies have shown that alexithymia has its own heritability component that cannot be fully explained by depression or a genetic susceptibility to general distress and psychopathology [76]. Consequently, although the evidence is mixed, it does suggest that alexithymia, although significantly related to depression, is an independent construct that needs to be considered separately from depression.

From a developmental perspective, structural equation modeling has suggested that childhood maltreatment has an indirect influence on eating disorder symptomatology such that the association between childhood maltreatment and eating symptomatology is mediated by alexithymia and depression, although there are some sex and cultural differences in these relations [65-69]. The identification of children with a history of childhood maltreatment and early intervention focused on depressive symptoms and the identification and communication of emotions may act as a protective factor and decrease the risk for the development of later disordered eating.

With respect to treatment, alexithymia levels decrease significantly in response to psychological treatments that place an emphasis on identifying and describing emotions in addition to symptom reduction [27,71-75]. However, despite these significant decreases in alexithymia scores from pre- to post-treatment, alexithymia scores still remain elevated compared to control scores at post-treatment, suggesting that more intensive treatment focused on emotion regulation may be needed.

### Critical appraisal of the research

The research that has been conducted to date has a number of strengths, including the use of large clinical and non-clinical samples to cover the entire spectrum of eating disorder behaviors, and the use of psychometrically valid measures for the assessment of eating disorder behaviors and alexithymia. However, there are a number of limitations that should be taken into consideration when conducting future research on the role of alexithymia in eating disorders.

First, almost all of the studies to date have been cross-sectional in nature, thus making it difficult to determine the direction of causality. The treatment studies conducted to date, although more longitudinal in nature, are also limited by the fact that alexithymia was measured only at pre-treatment and at post-treatment, again raising questions about causality. For instance, do alexithymia scores decrease before eating disorder symptomatology? Does eating disorder symptomatology decrease before alexithymia? What is the relation between changes in depression scores and changes in eating disorder symptomatology and alexithymia scores? Are there specific aspects of the treatment protocol that facilitate the decrease of alexithymia scores? These important empirical questions remain unanswered.

The assessment of alexithymia, eating disorder symptomatology, and depression at multiple time-points during treatment would be helpful in understanding the sequence of change during treatment, as well as the factors that may contribute to the decrease in alexithymia scores during treatment. Furthermore, with regards to treatment, longer follow-up periods are required to examine the maintenance of changes in alexithymia scores over time. As mentioned previously, although alexithymia scores decrease significantly from pre to post-treatment, the post-treatment alexithymia scores are still elevated compared to controls. However, it is possible that alexithymia scores continue to decrease following the completion of treatment as patients generalize the skills learned in treatment. Thus, a study that included multiple follow-up assessments (i.e., 1-month, 3-months, 6-months, 12-months post-treatment) would elucidate the long-term effects of treatment on alexithymia scores.

Longitudinal family-based studies may also enhance our understanding of the role of alexithymia in eating disorders. Given that eating disorders have a genetic basis [77], offspring of parents with a history of eating disorders are at an increased risk for developing disordered eating. Thus, assessing alexithymia levels and eating patterns in children of patients with eating disorders over time may help to better define the role of alexithymia in the development of eating disorders.

In addition to the cross-sectional nature of the studies, another limitation of the research to date is the reliance on self-report measures of alexithymia. Across studies, alexithymia has been assessed through a self-report measure that requires participants to reflect on their ability to identify and describe emotions as well as their cognitive style. However, individuals who struggle with these skills may experience difficulties reflecting on their abilities and may not report accurately on their skills. In response to this challenge, a few studies have begun to use observer-rated and skills-based measures of alexithymia. For instance, Berthoz and colleagues [57] found significant relations between self-reported TAS-20 scores and scores on the Observer Alexithymia Scale completed by a selected family member in a sample of 75 females with a diagnosis of an eating disorder. Using a skills-based approach, Zonnevijlle-Bender and colleagues [34] found that females between the ages of 12 and 18 years who met DSM-IV diagnostic criteria for AN self-reported significantly higher scores on the TAS-20 compared to controls, and also performed significantly worse on an emotion recognition task that required them to label pictures of emotional facial expressions. In a later study, Zonnevijlle-Bender and colleagues [78] identified a similar pattern of results in adult females with AN.

As described above, some studies using skill-based assessments have found consistency across self-report and skills-based assessments of alexithymia. Others, however, have produced inconsistent findings. For example, despite significant group differences on the TAS-20, female patients with AN and controls did not differ on the Levels of Emotional Processing Scale (LEAS) [79,80], a task which requires participants to rate how they would feel in 20 different scenarios [39,81]. There is some suggestion that the LEAS may measure a different aspect of alexithymia than the TAS-20 because the LEAS is not significantly correlated with the TAS-20 difficulties identifying and describing emotions factors, but is significantly correlated with the externally-oriented thinking factor [81]. Thus, the lack of group differences on the LEAS may be accounted for by the fact that identifying and describing emotions appear to be the main emotional processing deficits in eating disorders. The discrepant findings between the LEAS and the TAS-20 highlight the need to more precisely define the specific constructs that are being assessed by the various measures of alexithymia, and to identify overlapping and unique features.

Research on alexithymia and eating disorders has primarily been conducted using female Caucasian samples. Thus, there is a paucity of research that considers sex differences, especially with regards to AN and BN, as well as variability due to ethnicity. The few studies that have considered sex and ethnicity, however, suggest that they may be important moderating variables that warrant further investigation [68,69].

From a theoretical perspective, it is important to consider whether the application of the term alexithymia to the emotion deficits observed in patients with eating disorders is accurate. As described in the introduction, the original conceptualization of alexithymia consisted of 4 characteristics: 1) difficulties identifying feelings and differentiating between feelings and bodily sensations; 2) difficulties describing feelings; 3) lack of fantasy; and 4) an externally-oriented thinking style. Based on the studies to date, individuals with eating disorders only endorse two out of four of the characteristics of alexithymia, although the lack of fantasy life factor has not been adequately assessed. It is important to consider whether applying the term alexithymic to patients with eating disorders is accurate based on the research to date, or whether it would be more accurate to highlight specific deficits in identifying and describing emotions.

Moreover, questions have been raised regarding the similarities and differences between alexithymia and interoceptive awareness. Interoceptive awareness has been defined as the ability to “recognize and accurately respond to emotional states” [82], p. 6, and it is typically assessed using the Interoceptive Awareness subscale of the Eating Disorder Inventory (EDI) [43]. The EDI defines low interoceptive awareness as “confusion and apprehension in recognizing and accurately responding to emotional states” [82], p. 6. Research has demonstrated that individuals with eating disorders have low levels of interoceptive awareness [83]. During the development of the TAS, Bagby and colleagues [13] incorporated 4 items from the Interoceptive Awareness Scale of the EDI [44] for the difficulties identifying feelings factor. Across studies, it has been shown that alexithymia and interoceptive awareness are significantly positively correlated, even when controlling for the overlapping questions [28,51]. The main factors that differentiate between alexithymia and interoceptive awareness are that alexithymia also focuses on cognitive factors, such as an externally-oriented cognitive style. However, as seen through the present review, individuals with eating disorders only have elevated scores on the difficulties identifying and communicating emotions factors. Therefore, based on the research to date, it appears that alexithymia and interoceptive awareness both refer to the same concept. Thus, consolidating the two lines of research may help in creating a more parsimonious explanation for emotional processing deficits in eating disorders.

### Strengths and limitations of this review paper

The current paper provides a thorough, comprehensive review of the literature on alexithymia and eating disorders. It is important, however, to note some of the methodological limitations of the current review paper. First, although limiting the review paper to studies that used the TAS to assess alexithymia enhanced our ability to compare results across studies, it is also a limitation, as the paper does not include studies using other self-report and behavioral measures of alexithymia. Second, only one database (i.e., PsycInfo) was used to identify relevant articles and the database search was limited to English language papers. Third, only one author extracted the articles from the database and appraised them. Thus, we do not have reliability data for the extraction and appraisal process. Despite these limitations, to the best of our knowledge, this is the first comprehensive review paper focused on alexithymia and eating disorders, and the findings of the paper have theoretical and clinical implications for our understanding of eating disorders.

### Clinical implications

Cognitive behavior therapy and interpersonal therapy are the most widely used and studied psychological treatments for eating disorders. Cognitive behavior therapy focuses on teaching behavioral strategies to help patients restore normal eating, identifying and understanding triggers for their eating disorder behaviors, and examining and modifying their cognitions related to food, shape, and weight [84,85]. Interpersonal therapy views interpersonal problems as underlying eating disorder symptoms and focuses on helping patients learn more effective strategies for dealing with interpersonal challenges, such as improving communication skills, modifying expectations in relationships, and identifying and modifying problematic interaction styles [86]. According to the National Institute for Health and Clinical Excellence guidelines [87], CBT is the first line treatment for BN and BED while IPT is a second line treatment for BN. To date, no one treatment has been identified as clearly superior for AN (see [88] for a review).

It is important to note, however, that even with CBT, fewer than 50% of patients with BN experience a full recovery and the outcomes are even worse for patients with AN [88]. Thus, a significant proportion of individuals with eating disorders are not being helped considerably by our current treatment approaches. Given the significant role of emotion dysregulation in eating disorders and the finding that alexithymia is a negative predictor of treatment outcome [89], a treatment approach that emphasizes identifying and coping with emotions, in combination with traditional CBT strategies for normalizing eating, may assist in improving treatment efficacy. One treatment approach that has emotion dysregulation as a key pillar to its theoretical orientation is dialectical behavior therapy (DBT) [90,91]. Dialectical behavior therapy combines CBT approaches with acceptance-based approaches [92] and suggests that maladaptive behaviors, such as self-harm, function to regulate negative emotions in individuals who lack emotion regulation skills [90]. Thus, from a DBT perspective, the behaviors that are considered maladaptive in eating disorders, such as bingeing, purging, restricting food intake, exercising excessively, and using laxatives/diuretics, are negatively reinforced, as they function to regulate emotions and decrease feelings of distress [93,94]. Consequently, helping patients develop more adaptive strategies to cope with their emotions should help patients improve their eating disorder behaviors.

Dialectical behavior therapy includes learning a number of strategies, often in group format, that are directly focused on increasing patients’ skills to adaptively cope with strong urges and emotions. These strategies include: mindfulness, emotion regulation, distress tolerance, and interpersonal effectiveness [91].

The empirical evidence on DBT for BED and BN is currently in its infancy. To date, two case studies [95-97], an uncontrolled group treatment study [98], two wait-list studies and one active control treatment study [93,98,99] have been conducted. Across these studies, there is evidence that DBT leads to significant reductions in binge eating and purging behaviors from pre-treatment to post-treatment in patients with BN and BED, and that these reductions are maintained at 6-month follow-up [93,95,98]. There is also evidence of significant reductions in the tendency to eat when feeling anxious, depressed, or angry in patients with BN [95], but not in patients with BED [93,98].

To date, no studies have examined DBT for AN. However, there is some very preliminary evidence for the effectiveness of various emotion-based treatments for AN, including emotion-focused therapy [100], cognitive remediation and emotion skills training [101], and emotion acceptance behavior therapy [102]. Below, we provide a brief overview of these newer treatment approaches, highlighting key findings with regards to changes in emotion regulation skills. A detailed review of these newer treatment approaches is beyond the scope of the current paper.

Dolhanty and Greenberg [100] describe a single case study involving a 24-year old female with severe AN-R who experienced significant reductions in alexithymia scores following emotion-focused therapy. Emotion-focused therapy views emotions as being essential in helping individuals to understand the world and to respond effectively to various demands, challenges, and situations [103]. However, when individuals are raised in an environment that models emotions as being unacceptable (e.g., children being told that they should not cry) or overwhelming (e.g., mothers becoming overwhelmed by their children’s anxiety) then people are at an increased risk for developing emotional difficulties due to an inability to identify and/or regulate their emotions. Thus, the goal of emotion-focused therapy is to help patients increase their awareness and expression of their emotions while learning to react to their emotions in a more adaptive and healthy way that will aid their personal growth and development [104,105].

There is also some evidence regarding the effectiveness of skills-based treatments for AN. Money and colleagues [101] developed a skills-based adjunct to inpatient treatment for AN focused on modifying cognitive rigidity and developing skills in identifying and regulating emotions. In a case study of an adult female patient with AN, Money and colleagues [101] showed that the treatment was acceptable to the patient and lead to a small reduction in alexithymia scores, especially with regards to difficulties with identifying feelings [101]. Another recently developed treatment for AN is emotion acceptance behavior therapy [102]. This therapeutic approach combines the standard components of treatment for AN (i.e., weight restoration, normalizing eating behaviors) with “psychotherapeutic techniques designed to increase emotional awareness, decrease emotional avoidance, and encourage resumption of valued activities and relationships outside the eating disorder” ([102], p. 422). A series of case studies has shown that this treatment is effective in reducing AN symptoms, increasing awareness and tolerance of emotions, and is also rated as acceptable by patients [102].

In conclusion, a number of different emotion-focused treatment protocols have been developed for eating disorders. The research on the majority of these protocols is still in its infancy and there is a need for more studies with larger sample sizes and controlled study designs to provide more insights as to the effectiveness of emotion-focused therapy for eating disorders, especially AN.

## Conclusion

In summary, there is a robust body of literature showing that individuals with eating disorders experience difficulties identifying and describing emotions. Given these affect-related difficulties, it is important that eating disorder treatment protocols also incorporate a focus on emotions and emotion regulation. Future research should aim to address some of the methodological limitations in the research to date, including sole reliance on self-report measures of alexithymia, lack of experimental studies, lack of consideration of sex and ethnic differences, and an exclusive focus on negative emotions. An improved understanding of the role of emotions in eating disorders will help to enhance treatment protocols and better understand the etiological and maintenance factors involved in eating disorders.

## Competing interests

The authors do not have any competing interests to report.

## Authors’ contributions

MN conceived the topic, conducted the literature review and drafted the manuscript. TM and SC helped with developing the topic, and drafting and editing the manuscript. All authors read and approved the final manuscript.
